# Mapping of a major QTL for salt tolerance of mature field-grown maize plants based on SNP markers

**DOI:** 10.1186/s12870-017-1090-7

**Published:** 2017-08-15

**Authors:** Meijie Luo, Yanxin Zhao, Ruyang Zhang, Jinfeng Xing, Minxiao Duan, Jingna Li, Naishun Wang, Wenguang Wang, Shasha Zhang, Zhihui Chen, Huasheng Zhang, Zi Shi, Wei Song, Jiuran Zhao

**Affiliations:** 1 Beijing Key Laboratory of Maize DNA Fingerprinting and Molecular Breeding, Maize Research Center, Beijing Academy of Agriculture and Forestry Sciences (BAAFS), Beijing, China; 2 Institute of Crops Research, Hunan Academy of Agricultural Sciences, Changsha, China

**Keywords:** Salt tolerance, QTL mapping, SNP, Plant height, Maize

## Abstract

**Background:**

Salt stress significantly restricts plant growth and production. Maize is an important food and economic crop but is also a salt sensitive crop. Identification of the genetic architecture controlling salt tolerance facilitates breeders to select salt tolerant lines. However, the critical quantitative trait loci (QTLs) responsible for the salt tolerance of field-grown maize plants are still unknown.

**Results:**

To map the main genetic factors contributing to salt tolerance in mature maize, a double haploid population (240 individuals) and 1317 single nucleotide polymorphism (SNP) markers were employed to produce a genetic linkage map covering 1462.05 cM. Plant height of mature maize cultivated in the saline field (SPH) and plant height-based salt tolerance index (ratio of plant height between saline and control fields, PHI) were used to evaluate salt tolerance of mature maize plants. A major QTL for SPH was detected on Chromosome 1 with the LOD score of 22.4, which explained 31.2% of the phenotypic variation. In addition, the major QTL conditioning PHI was also mapped at the same position on Chromosome 1, and two candidate genes involving in ion homeostasis were identified within the confidence interval of this QTL.

**Conclusions:**

The detection of the major QTL in adult maize plant establishes the basis for the map-based cloning of genes associated with salt tolerance and provides a potential target for marker assisted selection in developing maize varieties with salt tolerance.

**Electronic supplementary material:**

The online version of this article (doi:10.1186/s12870-017-1090-7) contains supplementary material, which is available to authorized users.

## Background

Elevated salt content in the soil leads to the suppression of plant growth and the reduction of productivity. About 6% of the land on earth and 20% of the total cultivated land worldwide are affected by high salt [1, 2]. In many areas, salinity problem is further aggravated by unsustainable agricultural practices. Salt stress causes ion and hyperosmotic imbalance in plants, which causes secondary oxidative damage. These changes occur at the molecular, cellular, and whole-plant levels, resulting in the plant growth arrest and death [3, 4]. Therefore, understanding the genetic architecture of salt tolerance in plant is of great significance for the selection, utilization, and breeding of salt tolerant varieties.

A major salt tolerance strategy in plant is to re-establish cellular ion homeostasis [4]. A high concentration of sodium ions (Na^+^) inhibits many key enzymes, so Na^+^ influx to the cell cytoplasm and organelles is sophisticatedly regulated. Therefore, tremendous efforts have been made to identify the transporters modulating the influx and efflux of Na^+^ in plant cells. Vacuolar Na^+^/H^+^ antiporters manage the compartmental Na^+^ in the vacuole to prevent Na^+^ toxicity in cytosol, which has been shown as a strategy in many naturally salt tolerant plants (halophytes) [5]. In addition to the control of Na^+^ influx, Na^+^/H^+^ antiporters on the plasma membrane are also important in exporting Na^+^ to maintain low Na^+^ concentration in the cytoplasm. In *Arabidopsis*, a salt overly sensitive (SOS) signal transduction pathway has been identified to mediate ion homeostasis and Na^+^ tolerance [6]. *SOS1* encodes a plasma membrane Na^+^/H^+^ antiporter, and *sos1* mutation renders *Arabidopsis* the extreme sensitivity to Na^+^ stress [7]. Besides the SOS-dependent pathway, other mechanisms have also been reported to play roles in salt tolerance of plants, including the accumulation of osmolytes to establish osmotic homeostasis and the increase of antioxidants to mediate oxidative protection [3, 4].

Quantitative trait locus (QTL) mapping has been applied to detect the genetic basis of salt tolerance in many plants [8], which provides valuable information for further map-based cloning of salt tolerance genes and marker-assisted selection (MAS) in crop breeding. Using restriction fragment length polymorphism (RFLP) markers and an F_2:3_ population derived from the cross between salt tolerant and salt sensitive rice varieties, two major QTLs explaining 48.5% and 40.1% of the total phenotypic variance (PVE) were detected in rice [9]. The QTL detection led to the cloning of the gene responsible for salt tolerance, *SKC1*, which encoded a high affinity K^+^/Na^+^ transporter [10]. Taking advantage of the amplified fragment length polymorphism (AFLP), RFLP and simple sequence repeat (SSR) markers, a QTL, *Nax1*, was identified on the Chromosome 2AL of durum wheat using an F_2_ and an F_2:3_ population, which accounted for 38% of the phenotypic variation, and the SSR marker closely linked to the QTL was proven to be useful for the MAS in the breeding program [11]. Later, the *Nax2* locus was discovered and a Na^+^-selective transporter gene *TmHKT1;5-A* was subsequently characterized in durum wheat [12]. In soybean, utilizing random amplified polymorphic DNA (RAPD), insertion-deletion (InDel), and SSR markers with an F_2:3_ population and recombinant inbred lines (RILs), a salt tolerance QTL was mapped within a 209-kb region on Chromosome 3 and its flanking markers were used for MAS in soybean breeding [13]. In maize, nine conditional QTLs for salt tolerance at the seedling stage were identified on Chromosomes 1, 3, and 5 using single nucleotide polymorphism (SNP) markers and F_2:5_ RILs, three of which explained more than 20% of phenotypic variation [14]. However, studies of QTLs for salt tolerance in maize are still very limited, and QTLs for salt tolerance has not been reported in mature field-grown maize.

In this study, we identified a major QTL for salt tolerance in mature maize grown in a saline field using a permanent double haploid (DH) population and high-density SNP markers, and two candidate genes harbored in this QTL might be involved in the SOS pathway. Our results not only shed light on the mechanism of salt tolerance in field-grown maize, but will also facilitate the breeding of maize varieties with salt tolerance.

## Methods

### Plant materials and treatment

The parental maize inbred lines, PH6WC and PH4CV, were obtained from DuPont Pioneer (Johnston IA, USA). A DH population consisting 240 lines derived from the F_1_ hybrid of PH6WC × PH4CV was developed by pollinating with the parthenogenetic-inducing line Jingkeyou006 to obtain the haploid plants, and then followed by artificial doubling with colchicine [15]. Jingkeyou006 was obtained from the Maize Research Center of Beijing Academy of Agriculture and Forestry Sciences.

The 240 DH lines and their parents PH6WC and PH4CV were used in field experiments. For the salt stress treatment, plant materials were planted in the saline field at Tongzhou (TZ, N39°41′49.70″, E116°40′50.75′ in Google Earth™), Beijing, China, in the spring of 2014, 2015, and 2016. Plants cultivated in the normal field at Changping (CP, N40°10′50.38″, E116°27′15.40″), Beijing, in the spring of 2014, 2015 and 2016 served as controls. All field experiments were performed in accordance with local legislation. At each location, the experiments were conducted in a randomized complete block design [16–18] with two replicates in each year. For each block, 20 plants of each DH line were planted for a whole row, with the row length of 5 m and the spacing between rows of 60 cm.

### Soil sampling and analyses

According to the five-point sampling method [19], soil samples were collected from five representative locations (upper left, upper right, lower left, lower right and the middle) of the fields in both TZ and CP for composition analysis. Total salt content was determined using the residue-drying method [20]. Soil pH was measured using a pH meter (Hach, Loveland, CO, USA), and Na^+^ content was determined by flame emission spectroscopy (PerkinElmer, Norwalk, CT, USA) [21].

### Phenotype analysis

At harvest stage, in the summer of 2014, 2015, and 2016, plant height of the DH lines and their parents in the saline field (SPH) and control field (NPH) were recorded. Plant height was measured from the top of the main inflorescence down to the ground. For 240 DH lines, five randomly selected plants in each row were measured as one replicate, and two replicates were performed each year. After collecting plant height data in TZ and CP, salt tolerance index (PHI) based on plant height for each line was calculated using the following formula, PHI = H_SPH_/H_NPH_, where H_SPH_ represents the average height of five mature maize plants of each DH line grown in TZ, H_NPH_ represents the average height of 10 mature maize plants (5 of each replicate) of the same line grown in CP.

### Linkage map construction and QTL identification

Total genomic DNA was extracted from leaves using the CTAB method and then the 240 DH lines were genotyped using the MaizeSNP3072 chip [22]. A comparative linkage map was constructed using the Kosambi function in JoinMap4 software with a minimum LOD score of 2.0. Composite interval mapping was carried out using Windows QTL Cartographer software V2.5 which was developed by the Department of Statistics, NCSU with a walk speed of 1.0 cM and the LOD threshold of 3.0.

### Candidate gene analysis

Salt tolerance-related genes previously characterized in other plant species, such as *AtSOS1* [7], *AtSOS2* [23], *AtSOS3* [24], *OsSKC1* [10], and *TmHKT1;5* [12], were employed to query the maize genome database (MaizeGDB, http://www.maizegdb.org/, B73 RefGen_v2) using the tool of local BLASTP with the e-value cutoff of 1e-4 [25]. The maize homologs fell in the confidence interval of the identified major QTL were considered as candidate genes associated with salt tolerance.

### qRT-PCR analysis

Seeds of PH6WC and PH4CV were surface sterilized with 1% NaClO for 10 min, following by rinsing with sterile water for three times. The resulting sterile seeds were sown in maize seedling identifying instrument (Chinese patent, patent number: ZL200920177285.0) according to its manufacturer’s instructions, and then were placed in a greenhouse under 12 h light/ 12 h dark at 25 °C with the light density of 150–180 μmol m^−2^ s^−1^ and the relative humidity of 70% [26]. Seeds were grown in sterile water for three days and then in the Hoagland’s nutrient solution (Phyto Technology Laboratories Co., Ltd., USA) which was replaced with fresh Hoagland’s solution for every 2 days. After 7 days, non-germinated seeds and seedlings exhibited abnormal growth were discarded and the remaining seedlings were treated with 100 mM NaCl at day 12. Shoot and root samples were collected in three biological replicates each with five seedlings, and were immediately frozen in liquid nitrogen for RNA extraction.

Total RNA of shoot and root was extracted using the TRIZOL reagent (Invitrogen, Carlsbad, CA, USA). After RNA was treated with RNase-free DNase1 (Fermentas, Thermo scientific, USA), reverse transcription was conducted using the PrimeScript™ II 1st strand cDNA Synthesis Kit (Takara Bio Inc., Shiga, Japan) according to the user’s manual. qRT-PCR was carried out using the QuantStudio™ 6 Flex (ABI Life Technologies, USA). Gene-specific forward and reverse primers were designed (Additional file 1) using the PrimerQuest tool on IDTDNA (http://sg.idtdna.com/Primerquest/Home/Index). The reaction was performed in the 20 μl PCR system using the SYBR Premix Ex TaqII (Takara Bio Inc., Shiga, Japan) according to the user’s manual. *ZmActin1* served as the internal reference and mRNA relative expression levels were calculated using the 2^-△△Ct^ method.

### Statistical analysis

Using the Graphpad Prism software (http://www.graphpad.com/), the average of total salt content, Na^+^ content, and pH in fields of TZ and CP was compared by t-test. Two-way ANOVA with Bonferroni test was carried out for plant height of two parental lines grown in two soil conditions as well as the gene expression analysis. The frequency distribution, linear regression, and correlation analysis of the average of SPH, NPH and PHI across three environments were determined with the non-linear Gaussian regression, linear regression and correlation function in the column analysis, respectively. The combined ANOVA of SPH, NPH and PHI for the DH population and the heritability of three traits were obtained using the ANOVA tool in IciMapping 4.1 program [27].

## Results

### Soil composition

**Fig. 1:**
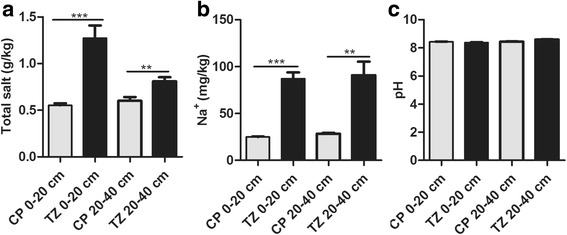
Soil composition in CP and TZ fields at depths of 0–20 and 20–40 cm. The bar charts represent the mean ± SE of five soil samples from each field. **a** Total salt content. **b** Na^+^ content. **c** pH value. ^*^, ^**^, and ^***^ indicate significant difference at *P* < 0.05, *P* < 0.01, and *P* < 0.001, respectively. CP indicates Changping. TZ indicates Tongzhou

Soil in agricultural land is usually subject to the compound effect of salt and alkali. To understand the main stress factor in soil in this study, we analyzed the soil composition in TZ and CP fields at the depths of 0–20 and 20–40 cm (Fig. 1). The total salt content and Na^+^ content of soil from both the 0–20 cm and 20–40 cm layers in TZ were significantly higher than those in CP, but no substantial difference in the pH was observed between two locations, indicating that salt, rather than pH, was the major stress factor in TZ soil.

### Phenotypic variation and correlations

**Fig. 2:**
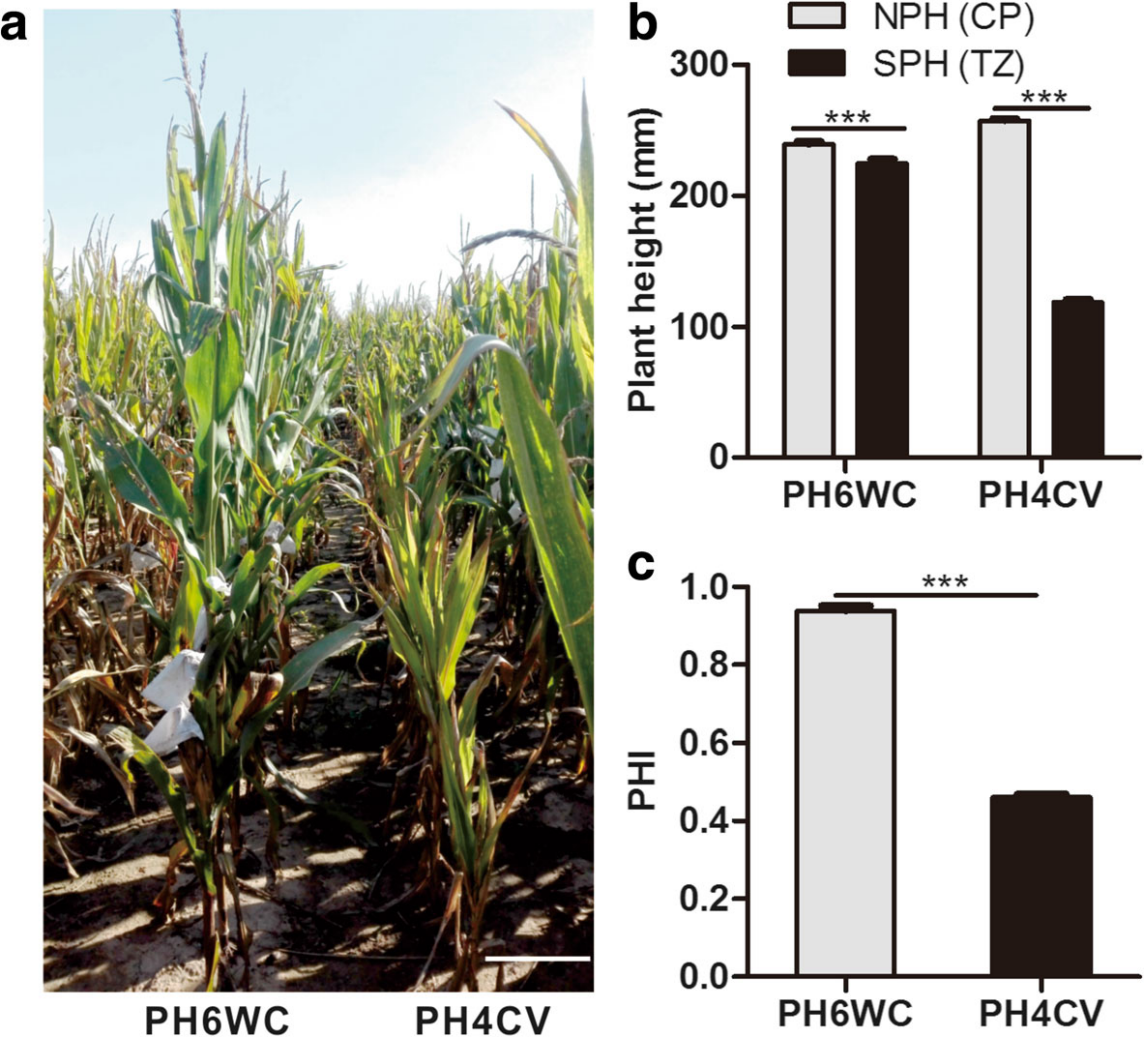
Comparison of mature maize plant height of PH6WC and PH4CV in saline field at TZ. **a** The representative image of field grown PH6WC and PH4CV in TZ. **b** Plant height of field grown PH6WC and PH4CV at Changping (CP) (control) and TZ (salt stress) in 2016. Bar charts represent the mean ± SE of 15 maize plants. **c** Effect of salt stress on plant height of PH6WC and PH4CV. Scale bar = 25 cm

**Fig. 3:**
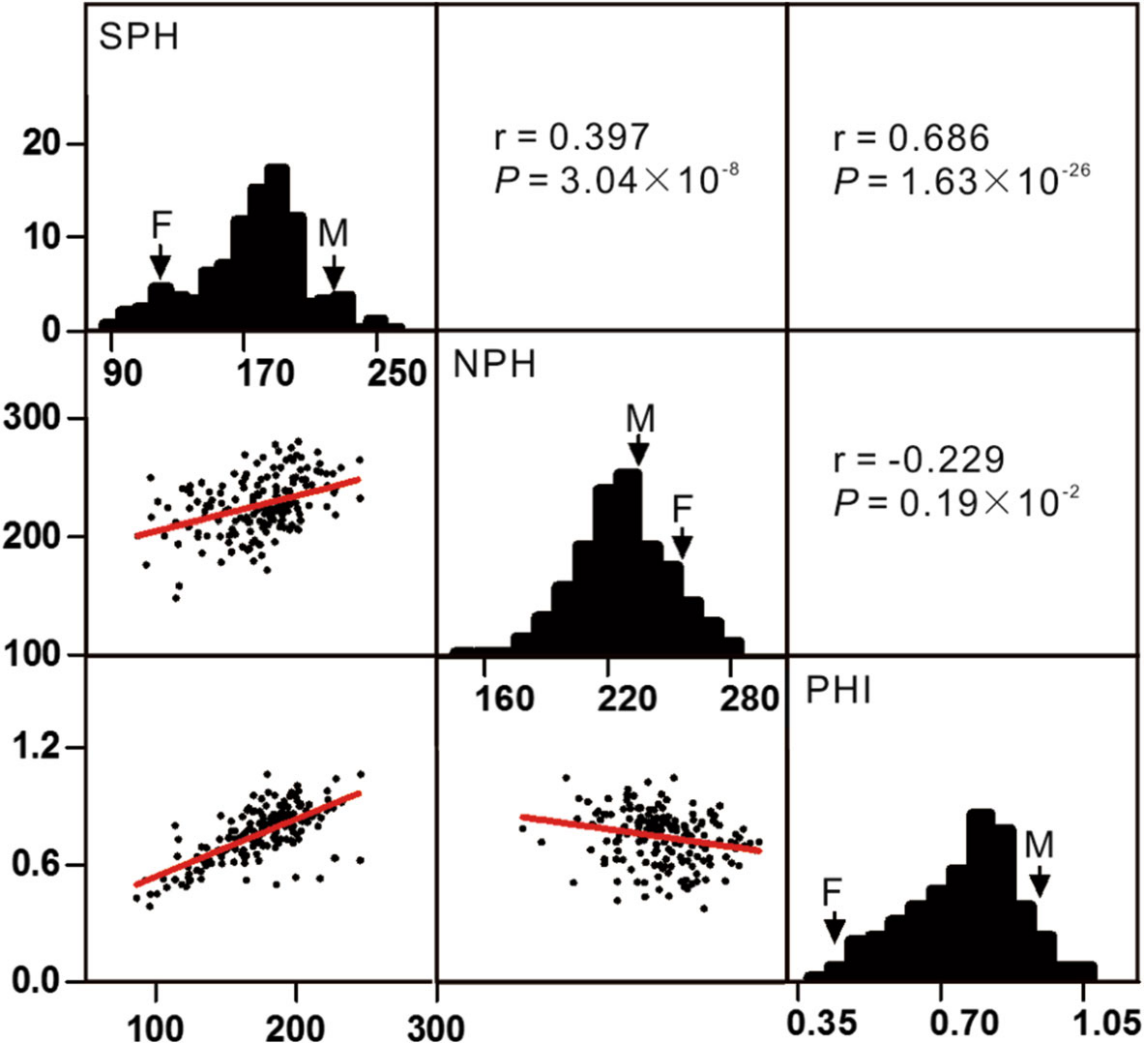
Frequency distribution and correlation analysis of NPH, SPH and PHI traits across three seasons. Frequency distribution and correlation analysis were conducted using Graphpad Prism software (http://www.graphpad.com/). r indicates correlation coefficient. F represents PH4CV and M represents PH6WC

To evaluate salt tolerance of mature maize in the field, plant height in the saline field (SPH) and salt tolerance index (PHI) were used as salt tolerance indicators. At the mature stage, although the plant height of both PH6WC and PH4CV were significantly reduced by salt stress (Fig. 2a and b), the PHI of PH6WC was significantly higher than that of PH4CV, indicating that PH6WC is less sensitive to salt stress compared to PH4CV (Fig. 2c). The variance effects of genotype (G), environment (E), and their interaction were extremely significant for all three traits (Table 1), and variance of the replicates within seasons for SPH and PHI was also significant. Broad sense heritability (*H* ^*2*^) of SPH, NPH and PHI was 74.7, 86.4 and 74.9% respectively, indicating that genotypes play an important role in the determination of these phenotypes. Phenotypic frequencies of all three traits showed a normal or near-normal distribution (Fig. 3). The correlation coefficient of the three-year average of SPH and NPH was low (*r* = 0.397), but it was as high as 0.686 between SPH and PHI, suggesting that the phenotypic results of SPH and PHI were highly positively correlated. As expected, NPH was negatively correlated with PHI with a low correlation coefficient of −0.229 (Fig. 3).

**Table 1:** Analysis of variance (ANOVA) for plant height of DH population in three environments

Trait	Source of variation	F	*H* ^*2*^
SPH	Environment (E)	31.8862^***^	0.7471
	Genotype (G)	14.1894^***^	
	Replication	3.0553^*^	
	G × E	4.4658^***^	
NPH	Environment (E)	4.7790^**^	0.8639
	Genotype (G)	39.2024^***^	
	Replication	0.7548	
	G × E	6.0168^***^	
PHI	Environment (E)	12.3846^***^	0.7492
	Genotype (G)	16.2502^***^	
	Replication	3.6290^*^	
	G × E	5.1059^***^	

*H* ^*2*^ indicates broad-sense heritability. ^*^, ^**^ and ^***^ represent significant levels at *P* < 0.05, *P* < 0.01 and *P* < 0.001, respectively

### Genetic linkage map construction based on SNP markers

A genetic linkage map was developed using the 240 DH lines and MaizeSNP3072 chip [22]. Of the 3072 SNPs, 1337 were polymorphic between the parents. For the 1337 SNPs, twenty SNPs were removed because of inconsistencies between their physical and genetic positions. With the 1317 SNPs, the linkage map constructed covered 10 chromosomes and spanned 1462.05 cM of the maize genome with an average distance of 1.11 cM between marker loci.

### Identification of salt tolerance related QTLs in maize

**Fig. 4:**
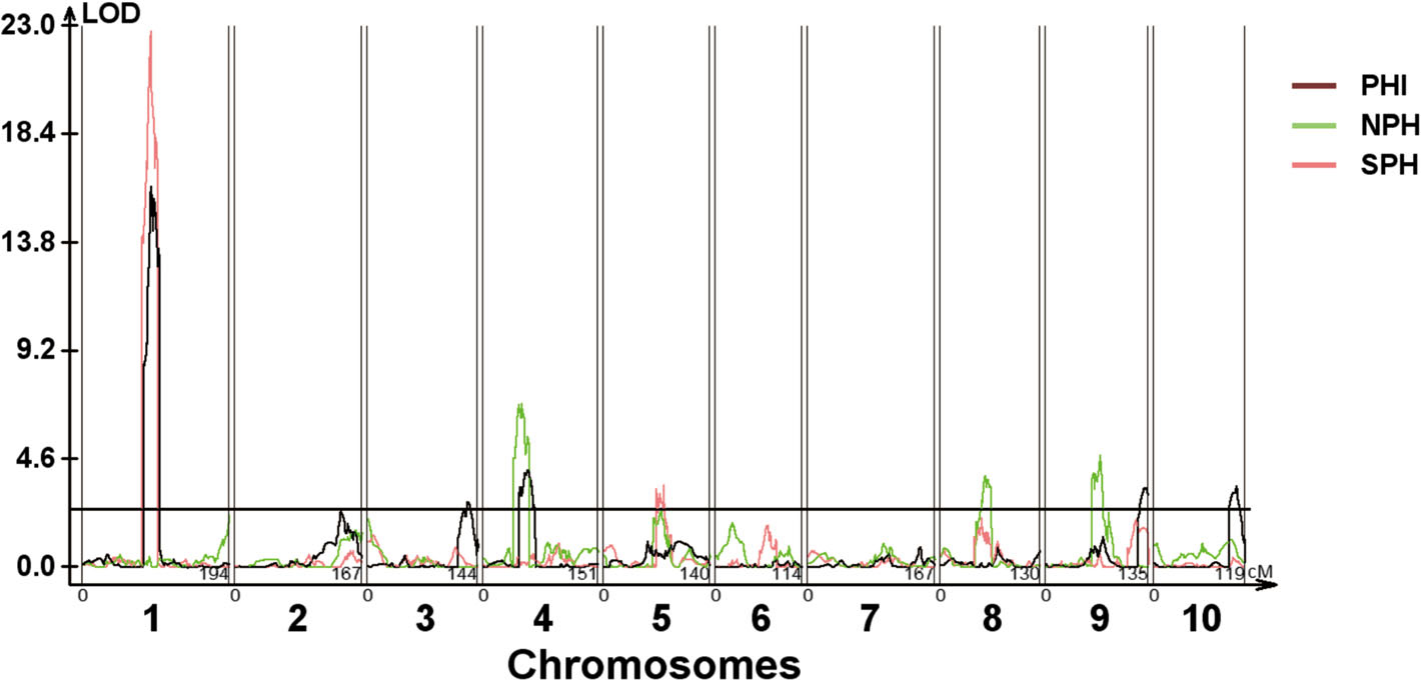
QTL locations of salt tolerance in field grown maize. *qSPH* represents QTL based on average plant height under salt stress across three seasons; *qNPH* represents QTL based on average plant height under normal condition across three seasons; *qPHI* represents QTL based on the average plant height salt tolerance index across three seasons

Based on the average plant height across three seasons at TZ, we identified one major QTL on Chromosome 1 with a LOD score of 22.40 and the PVE of 31.24%, which was designated as *qSPH1* (Table 2; Fig. 4). The LOD peak of *qSPH1* was mapped at 88.51 cM on Chromosome 1 and its confidence interval covered the region of 77.61–98.18 cM between the SNP markers *PZE101094436* and *PZE101150513*. In fact, *qSPH1* was detected across all seasons (Additional file 2), indicating that it is highly stable and not sensitive to environmental factors. Five QTLs controlling NPH were detected on chromosomes 4, 5, 8 and 9, respectively, with the PVE ranged from 5.0% to 11.9%. Except for *qNPH8* (LOD, 3.88; PVE, 6.36) which was identified across all three environments, *qNPH4*, *qNPH5*, *qNPH9–1* and *qNPH9–2* were detected only in single or two environments (Table 2; Fig. 4). There was no QTL controlling NPH mapped on Chromosome 1, revealing that *qSPH1* is the locus associated with salt tolerance, but not the plant height of maize.

**Table 2:** QTLs controlling plant height of mature maize grown in saline soil in three planting seasons

Traits^a^	Years	QTL^b^	Chr.	Position (cM)	Marker interval (Coordinates)	LOD	PVE^c^ (%)	Add^d^
NPH	2014	*qNPH4*	4	49.71	*PZE104023902 - SYN4889* (27048623–39,360,273)	4.39	8.33	8.40
		*qNPH8*	8	56.01	*PZE108041337- PZE108090114* (66870236–146,632,672)	3.67	6.90	8.12
	2015	*qNPH4*	4	46.01	*PZE104023902 - PZE104081530* (27048623–155,939,590)	6.28	10.11	7.78
		*qNPH5*	5	73.51	*PZE105045981 - PZE105128581* (34001093–185,707,663)	3.19	4.98	5.55
		*qNPH8*	8	61.61	*PZE108028588 - PZE108103365* (26352213–158,556,937)	5.03	7.07	6.94
		*qNPH9–2*	9	71.31	*PZE109064469 - SYN27201* (107573682–139,631,117)	4.56	7.24	6.61
	2016	*qNPH8*	8	57.31	*PZE108041337 - PZE108097446* (66870236–152,623,958)	4.29	7.75	8.11
		*qNPH9–1*	9	45.81	*PZE109011840 - PZE109040519* (12582417–62,366,526)	5.79	10.74	8.93
	mean	*qNPH4*	4	49.71	*PZE104023902 - PZE104081530* (27048623–155,939,590)	6.97	11.90	8.13
		*qNPH8*	8	57.31	*PZE108028588 - PZE108090114* (26352213–146,632,672)	3.88	6.36	6.40
		*qNPH9–2*	9	71.31	*PZE109064469 - SYN27201* (107573682–139,631,117)	4.80	7.97	6.66
SPH	2014	*qSPH1*	1	95.21	*SYN309 - SYN25920* (91198154–194,798,124)	4.47	13.28	13.79
	2015	*qSPH1*	1	88.51	*PZE101094436 - PZE101150513* (92353978–194,525,458)	11.46	16.99	16.86
		*qSPH 5–1*	5	77.31	*SYN16675 - PZE105128581* (165361318–185,707,663)	3.50	5.01	8.82
	2016	*qSPH1*	1	90.21	*PZE101109084 - SYN25920* (116462939–194,798,124)	19.47	35.03	24.69
		*qSPH5–2*	5	71.01	*PZE105049283 - PZE105117757* (40588124–174,670,738)	4.26	6.17	19.86
	mean	*qSPH1*	1	88.51	*PZE101094436 - PZE101150513* (92353978–194,525,458)	22.40	31.24	19.14
		*qSPH5–1*	5	77.31	*SYN1390 - PZE105128581* (163779167–185,707,663)	3.32	3.96	6.55
PHI	2014	*qPHI1*	1	94.81	*PZE101109084 - SYN25920* (116462939–194,798,124)	8.94	28.10	0.10
		*qPHI3*	3	23.51	*SYN28626 - PZE103019163* (2813337–11,407,445)	4.63	13.64	0.67
	2015	*qPHI1*	1	90.21	*SYN5444 - SYN25920* (96632104–194,798,124)	10.59	19.51	0.08
	2016	*qPHI1*	1	90.21	*PZE101109084 - SYN25920* (116462939–194,798,124)	14.27	27.51	0.09
	mean	*qPHI1*	1	90.21	*PZE101109084 - SYN25920* (116462939–194,798,124)	16.20	25.94	0.07
		*qPHI4*	4	56.91	*SYN4889 - SYN4250* (39360273–170,439,029)	4.14	5.63	0.03
		*qPHI9*	9	130.34	*SYN24345 - SYN5732* (153690286–155,054,745)	3.39	5.59	0.03
		*qPHI10*	10	108.16	*PZE110100655- SYN19213* (144384594–147,029,157)	3.29	4.47	0.03

^a^NPH, plant height under normal condition (Changping, Beijing). SPH, plant height under salt stress (Tongzhou, Beijing). PHI, plant height-based salt tolerance index. ^b^QTLs are designated as “q” followed by trait name: “NPH” (plant height under normal field), “SPH” (plant height under salt stress) and “PHI” (plant height-based salt tolerance index), and then the chromosome number. ^c^Phenotypic variation explained by QTL. ^d^Additive effect of tolerance allele

In addition, to exclude the impact of inherited factor on plant height under salt stress, we compared the plant height between the saline and control fields for each maize line to obtain PHI. Using the average of PHI across three seasons, a major QTL, named *qPHI1*, was identified on Chromosome 1 with the LOD score of 16.2, which accounted for 25.94% of the total phenotypic variation (Table 2; Fig. 4). The LOD peak of *qPHI1* was mapped at 90.21 cM on Chromosome 1 and its confidence interval spanned from 80.11 to 100.71 cM of the genetic map between the SNP markers *PZE101109084* and *SYN25920*. Similar to *qSPH1*, *qPHI1* was also identified across all three seasons (Additional file 2). The similar confidence intervals, positions, and LOD scores of *qSPH1* and *qPHI1*, along with the fact that no QTL detected for NPH on Chromosome 1 indicate that *qSPH1* is a major QTL responsible for the salt tolerance of mature maize but not the loci controlling the plant height.

Several minor QTLs were also detected under saline field condition, such as *qSPH5–1* and *qSPH5–2* on Chromosome 5. *qSPH5–2* (LOD, 4.26; PVE, 6.17%) was only identified in 2016, and *qSPH5–1* was detected both in 2015 and the mean of three-year SPH data (Table 2). Four additional minor QTLs responsible for PHI, named *qPHI3*, *qPHI4*, *qPHI9*, and *qPHI10*, were identified on Chromosomes 3, 4, 9 and 10, respectively, with the LOD score ranged from 3.29 to 4.63, which contributed 4.47% to 13.64% of the phenotypic variation. *qPHI3* was only detected in 2014, while other three minor QTLs were identified only based on the three-year average of PHI (Table 2). Their inconsistent detection in different planting seasons indicated that these QTLs were susceptible to environmental conditions.

### Candidate genes in the major QTL

*AtSOS1*, *AtSOS2*, *AtSOS3*, *OsSKC1*, and *TmHKT1;5* have been shown to play major roles in the salt tolerance of plants [4, 10, 12]. Using the protein sequences of these genes as queries, we screened the annotated maize genome database, and discovered two candidate genes within the confidence interval of the detected major QTL: *GRMZM2G007555* and *GRMZM2G098494* (Additional files 3 and 4). No homologs of *AtSOS2*, *OsSKC1*, and *TmHKT1;5* were identified within the QTL region. The product of maize gene *GRMZM2G007555* showed 61.38% identity to AtSOS3 which was predicted to encode a calcineurin B-like (CBL) protein, and thus it might be involved in regulating calcium signaling under salt stress. GRMZM2G098494 was a homolog of AtSOS1 and was annotated as a Na^+^/H^+^ antiporter.

**Fig. 5:**
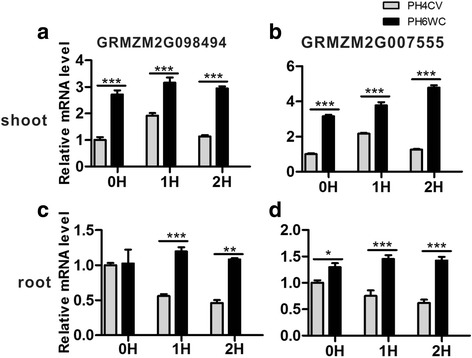
The relative expression level of potential candidate genes in maize seedlings of two *inbred lines*. Total RNA samples were collected at 0 h, 1 h and 2 h after salt treatment from shoot and root tissues, respectively. qRT-PCR was performed using maize *Actin* as the internal reference. Bar charts represent the mean ± SE of three biological replicates, each containing tissues from five individual seedlings. *Asterisks* show statistically significant difference in two lines by ANOVA. *, ** and *** indicate *P* < 0.05, *P* < 0.01 and *P* < 0.001, respectively. Expression of *GRMZM2G098494* in shoot (**a**), *GRMZM2G007555* in shoot (**b**), *GRMZM2G098494* in root (**c**) and *GRMZM2G007555* in root (**d**)

To further validate the potential candidate genes, their relative expression levels in the shoot and root of salt treated seedlings were determined by qRT-PCR (Fig. 5). In shoot, the expression of both candidate genes in the salt tolerant parent PH6WC was extremely significantly higher than that of the salt susceptible parent PH4CV at all three tested time points, 0 h, 1 h and 2 h after salt treatment, though the expression showed fluctuation in both lines (Fig. 5a and b). In root, although the basal expression of *GRMZM2G098494* in PH4CV was comparable to that in PH6WC, its induced expression was exceptionally higher in PH6WC compared to that of PH4CV (Fig. 5c). While the expression of *GRMZM2G07555* in PH6WC held at a steadily high level, it was dramatically decreased in PH4CV as the duration increase of salt treatment (Fig. 5d). Therefore, the detection of these two candidate genes in the major QTL along with their expression pattern upon salt treatment in both parents suggests that ion homeostasis regulation may play an important role in the salt tolerance of field-grown maize.

## Discussion

Utilizing salt tolerant maize germplasms is an important practice to battle the challenge of field saline stress, so understanding the genetic basis controlling salt tolerance in the mature field-grown maize can guide the breeding of maize varieties with salt tolerance. Therefore, QTL mapping for salt tolerance of mature maize in field conditions is of great significance, as it is closely related to crop production. In the present study, we identified a major QTL associated with salt tolerance in field-grown maize using a DH population (Table 2; Fig. 4).

Salt tolerance in plant is a complicated phenomenon and many parameters have been used as indicators to evaluate its effect, including agronomic traits [28], physiological indicators [26, 29] and phenotypic classification [14]. As an important index for plant salt tolerance, yield served as one of the phenotypic factors in the QTL analysis of salt tolerance in tomato [30, 31] and bread wheat [16]. However, grain yield in maize typically exhibits low heritability [32] and is affected by many external conditions. On the contrary, plant height is an important agronomic trait with relatively high heritability, and it has been shown to positively correlate to grain yield, such as in the V12 growth stage of maize [33], coffee [17] and wheat [34]. Therefore, along with its easy determination at the early growing stage, plant height may serve as a desired proxy of the grain yield. Actually, it has been employed in the identification of QTLs conditioning salt tolerance in other crop species, including a barley DH population [35] and rice RILs [36]. Thus, we believe that plant height of maize growing in the saline soil is the appropriate trait to investigate QTLs associated with salt tolerance.

In this study, the broad sense heritability of plant height in saline fields was 74.7%, indicating that phenotypic variation of this trait was largely determined by genotype. It is comparable with the high heritability of plant height in maize under heat stress [37] and the tall fescue under drought stress [38]. Based on this trait, a major QTL of *qSPH1* was identified on Chromosome 1, which explained 31.2% of phenotypic variation (Table 2). Only five minor QTLs controlling plant height with PVE less than 12% were identified under normal environment and none of them was co-localized with *qSPH1*, suggesting that the loci of *qSPH1* was linked to salt tolerance of mature field-grown maize.

To eliminate the inherent effect of agronomic traits, the ratio between stress and normal conditions has been used to evaluate the degree of stress tolerance in maize [39], rice [40], and cowpea [41]. Accordingly, the PHI for each DH line was calculated in this study and was used to map QTLs for salt tolerance. A major QTL, *qPHI1*, explaining 25.94% of PVE was identified at the same location as *qSPH1*, further verifying that this main QTL is responsible for salt tolerance in maize.

Although seedlings and mature plants are different at developmental stages, and their physiological characters and stress resistance mechanisms usually vary [42], but the genetic factors controlling a certain trait can still be the same in different stages [43]. Using an F_2:5_ RILs population, Cui et al., identified a major QTL, *QFgr1* or *QFstr1*, flanked by the marker *PZE101140869* and *PZE101138116*, which was responsible for the maize germination rate in saline fields or seedling salt tolerance ranking [14]. Comparing the QTL locations, we found that the position of *qSPH1* and *qPHI1* was comparable to *QFgr1* or *QFstr1*. Thus, it seems that this major QTL confers salt tolerance in two different populations, and it controls salt tolerance of both maize seedling stage and mature stage. In this regard, this QTL location may serve as a good target site for MAS in maize breeding to increase the salt tolerance of salt susceptible and elite inbred lines during both seedling and adult stages.

Tremendous efforts have been made to identify genes controlling salt tolerance in plants. In *Arabidopsis*, the SOS pathway has been demonstrated to mediate salt tolerance which comprises three members: SOS1, SOS2, and SOS3. After binding Ca^2+^, SOS3 interacts with SOS2 to form a kinase complex, which further activates SOS1, a plasma membrane Na^+^/H^+^ antiporter that exports Na^+^ out of the cell [3, 4]. In addition, *OsSKC1* [10] and *TmHKT1;5* [12] were responsible for salt tolerance in rice and wheat, respectively, and they were both cloned as Na^+^ transporters. To investigate whether genes in the confidence interval of the major QTL in the present study share any similarity to these genes cloned in other species, blastp was performed and two proteins GRMZM2G007555 and GRMZM2G098494 were shown to be homologous to AtSOS3 and AtSOS1 with the e-value of 1.740e-62 and 0, respectively. Such low e-values showed substantial protein identity, suggesting that two candidate genes may share similar function to *AtSOS3* and *AtSOS1* in the resistance to salt stress. The induced level of both genes was not substantially elevated at 1 h and 2 h after salt treatment (Fig. 5), which probably due to the sample collecting time points and the possible discrepancy in the salt response time between *Arabidopsis* [44] and maize seedlings, but the expression of both *GRMZM2G098494* and *GRMZM2G007555* in the salt tolerant PH6WC was significantly higher than that of PH4CV in both shoot and root tissues of maize seedlings in basal and induced conditions, except for *GRMZM2G098494* at 0 h in root. In addition, the expression pattern of both genes in PH4WC was declined as the increase in the treatment duration, whereas PH6WC exhibited a stable or slightly increased level, suggesting that these two genes may be associated with the difference in salt tolerance of both parents. Further investigation with more time points is required to fully understand their expression in the course of salt treatment. Therefore, we speculate that the regulation of ion homeostasis by the SOS pathway may be the genetic control for salt tolerance of mature field-grown maize. However, further research is needed to verify the function of these genes in salt tolerance, which will potentially facilitate the breeding of salt tolerant maize varieties.

## Conclusions

In the present study, we have constructed a SNP-based maize genetic linkage map and mapped a major QTL at the 88–95 cM region on Chromosome 1 based on plant height of mature maize grown in a saline field, which accounted for 31.24% of PVE. This main effect QTL for salt tolerance was further validated by QTL mapping based on plant height salt tolerance index, and two candidate genes with predicted functions in the SOS pathway have been identified in the locus, indicating that ion homeostasis regulation may play an important role in the salt tolerance of mature maize. Therefore, this locus will be valuable for marker assisted selection in maize breeding.

## Additional files

Additional file 1: Table S1. The primers used in the qRT-PCR. (DOCX 15 kb)

Additional file 2: Figure S1. Chromosomal locations and logarithm of odds (LOD) scores of the major QTL conditioning SPH and PHI on Chromosome 1 using data from individual years and overall means. Mean represents the average of three growing seasons. (DOCX 1512 kb)

Additional file 3: Figure S2. Protein sequences alignments by BLAST against B73 filtered gene set translations 5b.60 for RefGen_v2 (maizesequence.org) database. **(a)** Alignment of AtSOS1 and GRMZM2G098494. **(b)** Alignment of AtSOS3 and GRMZM2G007555. (DOCX 10769 kb)

Additional file 4: Table S2. The identity of two candidate genes identified in the major QTL region to *Arabidopsis SOS* genes and their positions and annotations. (DOCX 22 kb)

## Abbreviations

AFLP: Amplified fragment length polymorphism
CBL: Calcineurin B-like
CP: Changping
DH: Double haploid
E: Environment
G: Genotype
*H*^*2*^: Broad sense heritability
InDel: Insertion-deletion
LOD: Logarithm of odds
MAS: Marker-assisted selection
Na^+^: Sodium ions
NPH: Plant height in control field
PHI: Salt tolerance index
PVE: Phenotypic variance
QTL: Quantitative trait loci
RAPD: Random amplified polymorphic DNA
RFLP: Restriction fragment length polymorphism
RILs: Recombinant inbred lines
SNP: Single nucleotide polymorphism
SOS: Salt overly sensitive
SPH: Plant height in the saline field
SSR: Simple sequence repeat
TZ: Tongzhou
