# Supplementary material for: Mapping of a major QTL for salt tolerance of mature field-grown maize plants based on SNP markers
Source: BMC Plant Biol. 2017 Aug 15;17:140. doi: 10.1186/s12870-017-1090-7 (PMC5556339; doi:10.1186/s12870-017-1090-7)
Supplement: Supplementary file 1 — The primers used in the qRT-PCR. (DOCX 15 kb) [file 12870_2017_1090_MOESM1_ESM.docx]

| **Gene name** | **Protein name** | **F/R** | **5'--3' Primers** |
| --- | --- | --- | --- |
| GRMZM2G126010 | actin 1 | F | GTACCCGATTGAGCATGGCA |
|  |  | R | ACAGGGTGATCTTCAGGCGA |
| GRMZM2G098494 | putative protein | F | CGTGCTCACAGAATCGCAAG |
|  |  | R | TTCCTTCTCTTCCAGCAGCC |
| GRMZM2G007555 | putative protein | F | GCTCGTGCTCTCTCAGTATTT |
|  |  | R | CCTCTTGTCTCTCAATGTAGCC |

**Additional file 1: Table S1** The primers used in the qRT-PCR.
