# Supplementary material for: Mapping of a major QTL for salt tolerance of mature field-grown maize plants based on SNP markers
Source: BMC Plant Biol. 2017 Aug 15;17:140. doi: 10.1186/s12870-017-1090-7 (PMC5556339; doi:10.1186/s12870-017-1090-7)
Supplement: Supplementary file 2 — Chromosomal locations and logarithm of odds (LOD) scores of the major QTL conditioning SPH and PHI on Chromosome 1 using data from individual years and overall means. Mean represents the average of three growing seasons. (DOCX 1512 kb) [file 12870_2017_1090_MOESM2_ESM.docx]

**
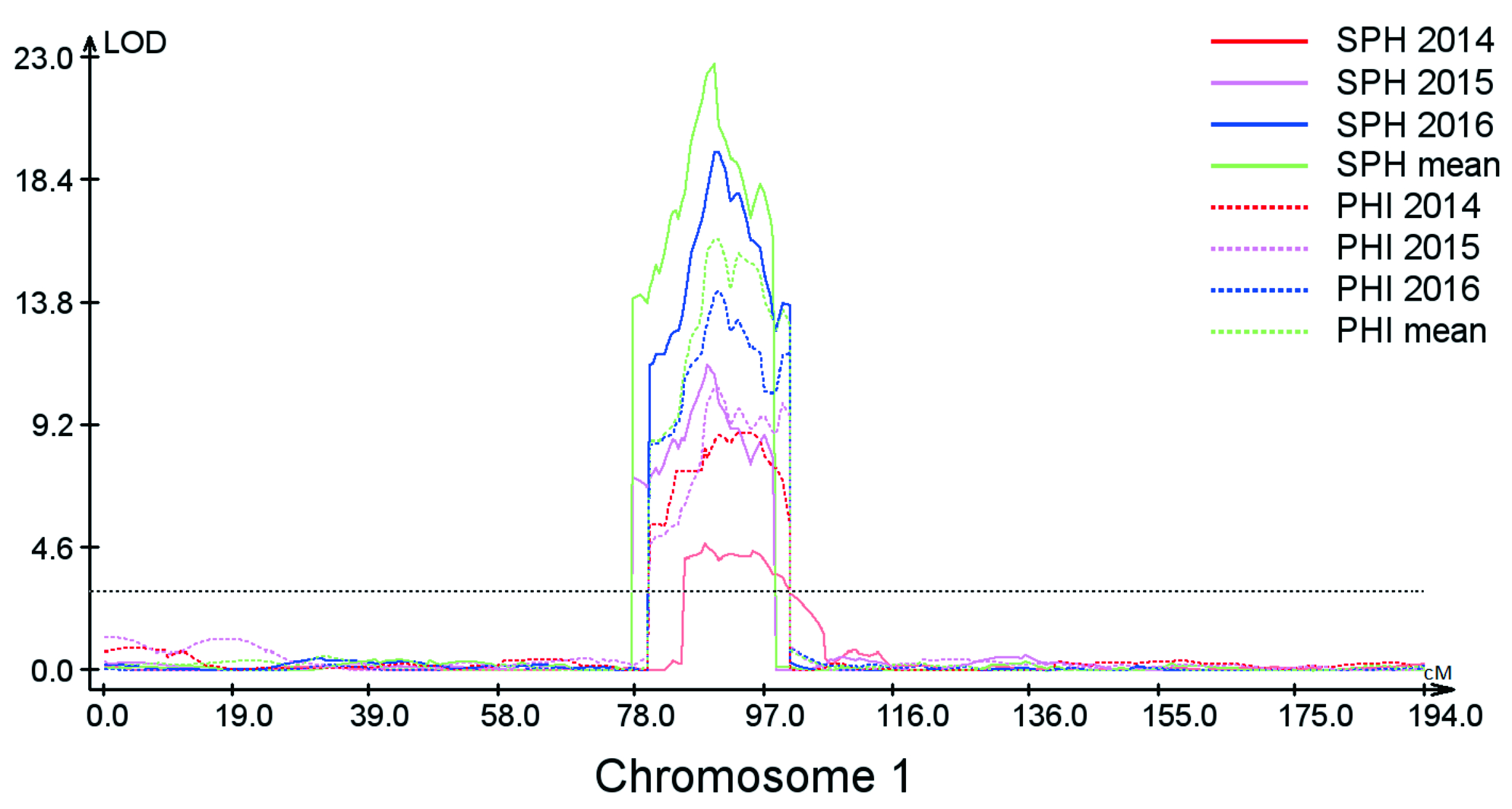
**

**Additional file 2: Figure S1** Chromosomal locations and logarithm of odds (LOD) scores of the major QTL conditioning SPH and PHI on Chromosome 1 using data from individual years and overall means. Mean represents the average of three growing seasons.
