# Supplementary material for: Mapping of a major QTL for salt tolerance of mature field-grown maize plants based on SNP markers
Source: BMC Plant Biol. 2017 Aug 15;17:140. doi: 10.1186/s12870-017-1090-7 (PMC5556339; doi:10.1186/s12870-017-1090-7)
Supplement: Supplementary file 3 — Protein sequences alignments by BLAST against B73 filtered gene set translations 5b.60 for RefGen_v2 (maizesequence.org) database. (a) Alignment of AtSOS1 and GRMZM2G098494. (b) Alignment of AtSOS3 and GRMZM2G007555. (DOCX 10769 kb) [file 12870_2017_1090_MOESM3_ESM.docx]

**
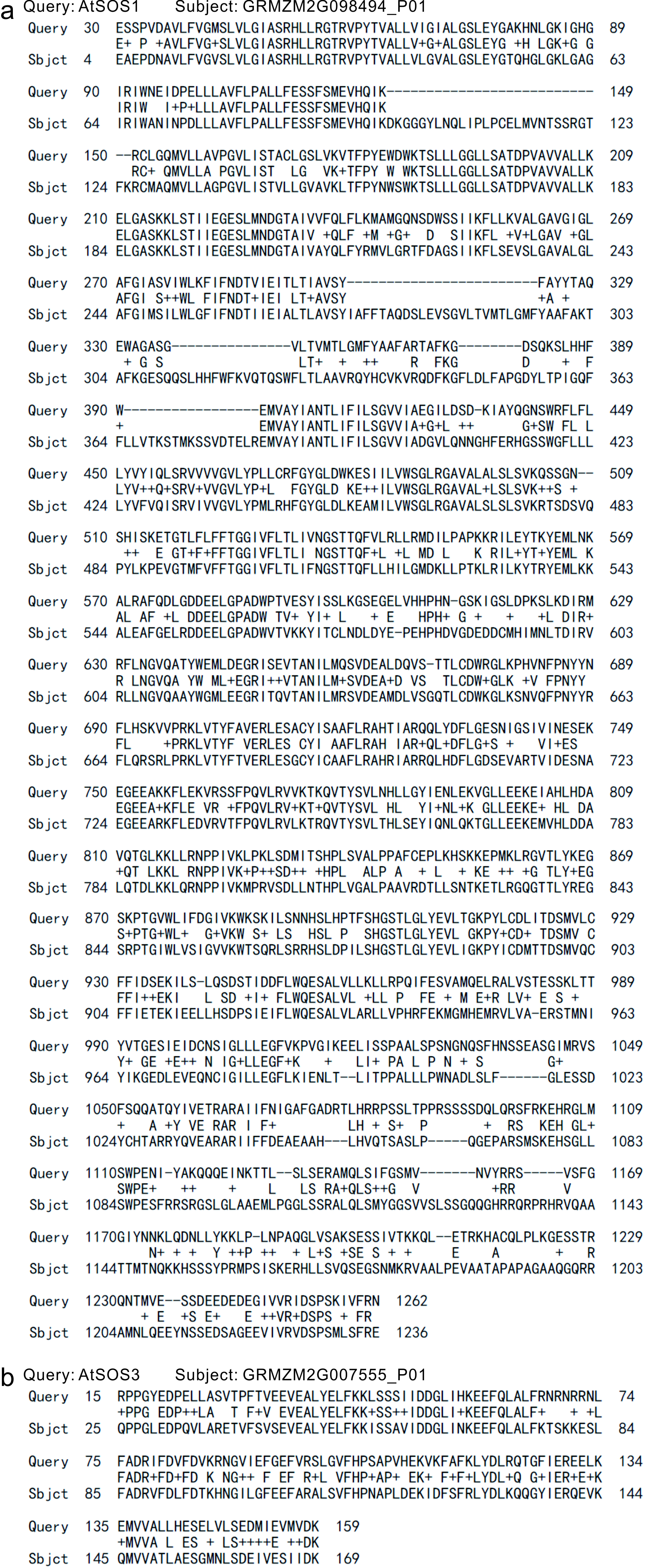
**

**Additional file 3: Figure S2** Protein sequences alignments by BLAST against B73 filtered gene set translations 5b.60 for RefGen_v2 (maizesequence.org) database. **(a)** Alignment of AtSOS1 and GRMZM2G098494. **(b)** Alignment of AtSOS3 and GRMZM2G007555.
