# Supplementary material for: Mapping of a major QTL for salt tolerance of mature field-grown maize plants based on SNP markers
Source: BMC Plant Biol. 2017 Aug 15;17:140. doi: 10.1186/s12870-017-1090-7 (PMC5556339; doi:10.1186/s12870-017-1090-7)
Supplement: Supplementary file 4 — The identity of two candidate genes identified in the major QTL region to Arabidopsis SOS genes and their positions and annotations. (DOCX 22 kb) [file 12870_2017_1090_MOESM4_ESM.docx]

**Additional file 4: Table S2** The identity of two candidate genes identified in the major QTL region to *Arabidopsis SOS* genes and their position and annotation.

| Query | Subject | Position | Annotation | Ident (%) | E value |
| --- | --- | --- | --- | --- | --- |
| AtSOS1 | GRMZM2G098494 | Chr1(180935605-180953553) | Na^+^/H^+^ antiporter | 53.37 | 0.0 |
| AtSOS3 | GRMZM2G007555 | Chr1 (167197487-167207198) | Calcineurin B-Like (CBL) protein | 61.38 | 1.740e-62 |
